# Doxorubicin induces an extensive transcriptional and metabolic rewiring in yeast cells

**DOI:** 10.1038/s41598-018-31939-9

**Published:** 2018-09-12

**Authors:** Hilal Taymaz-Nikerel, Muhammed Erkan Karabekmez, Serpil Eraslan, Betül Kırdar

**Affiliations:** 1 Department of Genetics and Bioengineering, Istanbul Bilgi University, 34060 Eyup, Istanbul Turkey; 2 Department of Chemical Engineering, Bogazici University, 34342 Bebek, Istanbul Turkey; 3 Department of Bioengineering, Istanbul Medeniyet University, 34000 Kadikoy, Istanbul Turkey; 4 Koç University Hospital, Diagnosis Centre for Genetic Disorders, Topkapı, Istanbul Turkey

## Abstract

Doxorubicin is one of the most effective chemotherapy drugs used against solid tumors in the treatment of several cancer types. Two different mechanisms, (i) intercalation of doxorubicin into DNA and inhibition of topoisomerase II leading to changes in chromatin structure, (ii) generation of free radicals and oxidative damage to biomolecules, have been proposed to explain the mode of action of this drug in cancer cells. A genome-wide integrative systems biology approach used in the present study to investigate the long-term effect of doxorubicin in *Saccharomyces cerevisiae* cells indicated the up-regulation of genes involved in response to oxidative stress as well as in Rad53 checkpoint sensing and signaling pathway. Modular analysis of the active sub-network has also revealed the induction of the genes significantly associated with nucleosome assembly/disassembly and DNA repair in response to doxorubicin. Furthermore, an extensive re-wiring of the metabolism was observed. In addition to glycolysis, and sulfate assimilation, several pathways related to ribosome biogenesis/translation, amino acid biosynthesis, nucleotide biosynthesis, *de novo* IMP biosynthesis and one-carbon metabolism were significantly repressed. Pentose phosphate pathway, MAPK signaling pathway biological processes associated with meiosis and sporulation were found to be induced in response to long-term exposure to doxorubicin in yeast cells.

## Introduction

Doxorubicin, a natural anthracycline antibiotic, is one of the most effective chemotherapy drugs used against solid tumors in the treatment of several cancer types. Like other anthracyclines, it is presumed to interact with DNA through intercalation, but the mechanism is not fully known^1^. One of the mechanisms proposed to explain the effect of doxorubicin on cancerous cells is the intercalation into DNA and thus leading to disruption of DNA repair, which would be mediated by topoisomerase II. Doxorubicin inhibits topoisomerase II, which overwinds DNA during transcription, thereby preventing the recombination of the DNA double strand, thus stopping DNA replication^2^. Another proposal is the generation of free radicals, which in turn might damage DNA and result in cell death^3^. There is also evidence on the enhancement of nucleosome turnover around promoters by doxorubicin, attributed due to its intercalation property^4^. These alterations in the nucleosome assembly are believed to affect mechanisms of cell killing during chemotherapy.

Research has been conducted on different types of cancer cell lines and cell models, with various concentrations of doxorubicin. Despite the fact that the responses of different cell types at transcriptional level show variations^5,6^, doxorubicin was found to play role in some additional cellular processes, including ceramide metabolism^7^ and cell cycle arrest^8^.

Resistance to chemotherapeutic agents is one of the major disadvantages of long-term anticancer treatment. Repeated doxorubicin administration leads to drug-resistant cancer cells and increased cytotoxicity^9^. In fact, cardiotoxicity is the most common doxorubicin-induced side effect. Therefore, most of the research conducted on doxorubicin has been focused on the elimination of the anti-therapeutic effects. Potential treatment options have been developed to reduce doxorubicin-mediated cardiotoxicity, such as lowering the dosage of doxorubicin, combined therapies with cardioprotective agents (e.g. dexrazoxane)^10^, through regulation of cardiac circular RNA expression^11^. The former approach of modulating the drug dosage was put forward, aiming to diminish the significant effects of oxidative stress, which is the major cause of cardiotoxicity, but subsequent studies revealed that minimizing or eliminating reactive oxygen species (ROS) did not solve the detrimental effects^12^. The underlying mechanisms of the cytotoxic side-effects are still not clear. Therefore, new approaches are required in order to prevent cardiotoxicity. Recent studies have already been started targeting the mechanisms causing apoptosis^13,14^.

Given the above-mentioned arguments, there has not been any unifying conclusion on the working mechanism of doxorubicin and its effects on cell metabolism are not fully established. Although several studies were designed to understand the mechanism of doxorubicin, most are focused on individual genes, whose expression has changed in the presence of doxorubicin, and ignored the system level genome-wide interactions in cells^15–19^. However, valuable information may be obtained through the integration of high-throughput -omics measurements at different levels with known metabolic/regulatory/interaction networks^20^, which may then be applied in studies for personalized medicine^21^.

Since the genes affected by chemotherapeutic drugs are well protected among eukaryotes, *Saccharomyces cerevisiae*, a eukaryotic model organism suitable to study the effects of chemicals on living cells at -omics level, has been an important tool for cancer research^22,23^. *S*. *cerevisiae* has been used to study the effects of doxorubicin on growth inhibition^24^, on cell cycle^25^, on DNA double-strand breaks^26^ and on environment-dependent protein complex dynamics^27^. Genome-wide screening with haploid and diploid *S*. *cerevisiae* deletion collections to identify the genes that induce doxorubicin resistance has also provided valuable information^28,29^. However, a systematic study using different approaches under similar conditions has been overlooked.

In the present study, an integrative systems biology approach was used to elucidate the long-term effect of doxorubicin in *S*. *cerevisiae* cells. Analysis and integration of the fluxomic data with transcriptomic response of yeast cells, which were grown and exposed to doxorubicin in well-controlled bioreactors, revealed an extensive rewiring of transcription and metabolism.

## Results

Genome-wide transcriptomic response of yeast cells to the long-term presence of doxorubicin was analysed in the samples collected at the mid-exponential phase growth and integrated with the flux-balance analysis, followed by protein-protein interaction network in order to identify the long-term effect of the drug on living cells. The cells were grown in triplicates in batch cultures in the presence or absence of doxorubicin.

### Fermentation characteristics of yeast cells

The preliminary shake flask experiments carried out in the presence of changing concentrations of doxorubicin (5 to 50 µM) indicated a decreasing growth rate with increasing concentrations of the drug. 20 µM doxorubicin, which was found to decrease the maximum growth rate from 0.3 h^−1^ to 0.1 h^−1^, was selected for further experiments (Supplementary Fig. S1). Yeast cells were shown to retain their >80% viability at this concentration^28^.

The cultures grown in the absence or presence of doxorubicin in bioreactors were characterized for their uptake/secretion rates during the mid-exponential phase. When the mass-balance-based biomass-specific rates for both conditions were compared, it was observed that the maximum growth rate was decreased from 0.27 ± 0.01 h^−1^ to 0.10 ± 0.01 h^−1^ in the presence of doxorubicin (Supplementary Table S1), as was the case in shake flask experiments. Since the growth rate was lower, glucose uptake, glycerol and ethanol production rates were also lower in the cultures grown in the presence of doxorubicin (Supplementary Table S1).

### Intracellular Fluxes in the presence of doxorubicin

The fluxes through the metabolic network of *S*. *cerevisiae* were calculated for growth on glucose for the cultures grown in the absence and presence of doxorubicin. The rates presented in Table S1 were used as inputs, and model was solved by optimizing the maximization of the ATP production, to estimate the fluxes via flux balance analysis. Among 3498 reactions within the model, 278 reactions were different based on their magnitude of change (down: <0.8, up: >1.10). 260 fluxes were decreased, whereas 18 fluxes were increased in the presence of doxorubicin, compared to the control cultures (Supplementary Table S2).

**Figure 1:**
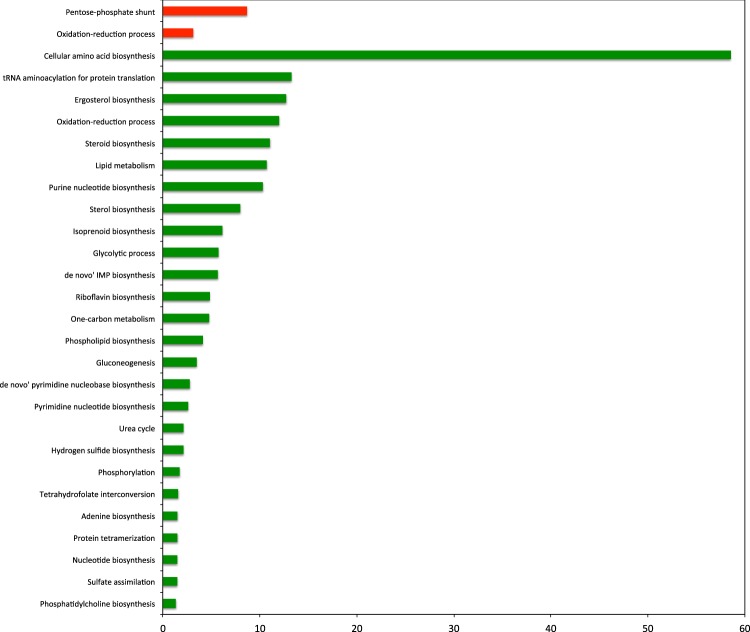
GO biological process terms associated to differential fluxes, changed in response to doxorubicin plotted against negative log values (base10) of corrected p-values. Red and green colors indicate increased and decreased fluxes, respectively.

The increased fluxes were mainly pentose phosphate (PP) pathway reactions and transport of O_2_. The decreased fluxes in the presence of doxorubicin were glycolytic reactions, transport reactions, and several reactions involved in amino acid biosynthesis (Supplementary Table S2). To find out the functional annotation biological process gene ontology (GO) terms of these reactions, the genes associated with the reactions of interest were identified. However, for the transport reactions the associations of gene-reaction were not provided. These gene sets (excluding transport) were used in GO-term enrichment analysis. The genes associated with the reactions, which showed increased flux values, were found to be mainly related to pentose phosphate shunt and oxidation-reduction process (Fig. 1). The genes associated with the reactions, which showed decreased fluxes, were found to be related to amino acid biosynthetic process, metabolic process, oxidation-reduction process, tRNA aminoacylation for protein translation among others (see Fig. 1 and Supplementary Table S3).

### Transcriptional response to doxorubicin

The analysis of genome-wide transcriptional response of yeast cells to long-term exposure to doxorubicin revealed that 1279 genes were differentially and significantly (a fold change greater than 1.5 and p value < 0.05) expressed when compared to control (Supplementary Table S4).

**Figure 2:**
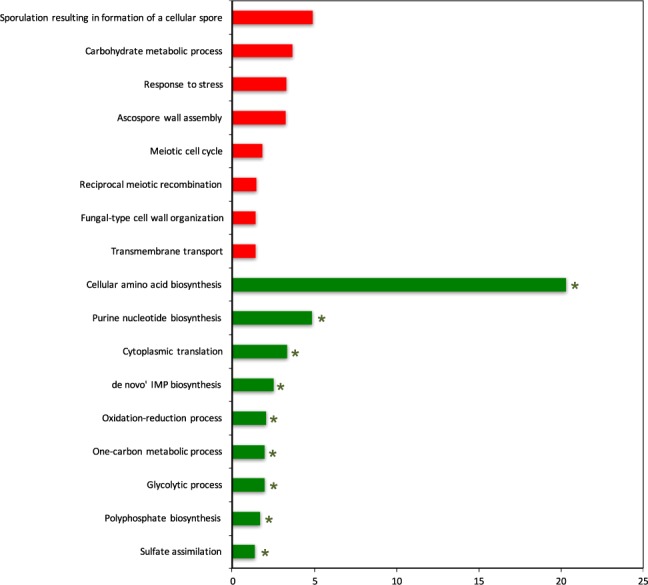
GO biological process terms significantly associated to genes differentially expressed in response to doxorubicin plotted against negative log values (base10) of corrected p-values. Red and green colors indicate up- and down-regulated biological processes, respectively. * indicates the terms that are also observed for differential fluxes (shown in Fig. 1).

A total of 976 genes were found to be up-regulated and GO biological process terms which were significantly (Benjamini-Hochberg corrected p value < 0.05) associated with 785 genes of this group excluding the genes with unknown biological function were identified (Supplementary Table S4). These genes significantly associated with biological processes such as sporulation resulting in formation of a cellular spore, response to stress, carbohydrate metabolic process, meiotic cell cycle, reciprocal meiotic recombination, fungal-type cell wall organization, and transmembrane transport displayed increased levels of expression when compared to control (Fig. 2, Supplementary Table S5). These genes were found to be significantly enriched in pathways related to starch, galactose, amino sugar and sucrose metabolisms, MAPK signaling pathway and meiosis.

A total of 35 transcription factors, which were mainly implicated in the regulation of cellular response to nutrient levels and general stress, were identified among the up-regulated genes. The major repressor of DNA damage regulated genes *(RFX1*), which is involved in DNA damage and replication checkpoint pathway, transcriptional factors involved in the regulation of metallothionein genes in response to DNA damage (*CUP2*) and in the pleiotropic drug resistance (*PDR8*, *PDR1* and *YRM1*) were also observed to be induced in response to exposure to doxorubicin. The higher expression levels of *RFX1*, *CUP2* and *PDR8* were also confirmed by RT-qPCR analysis (Supplementary Figs S2 and S3).

Manual investigation of up-regulated genes indicated that several genes involved in cellular response to oxidative stress (*TRR2*, *MHR1*, *ECM5*, *FRM2*, *HSP12*, *HSP31*, *GRX7*, *GPX1*, *SRX1*, *GCY1*, *MTL1*, *XBP1*, *TSA2*, *LOT6*, *MCR1*, *HBN1*, *VHR1*, *NTG1*, *NQM1*, *YJR096W*, *PRX1*, *GAD1*), siderophore and iron ion transport and homeostasis (*FIT3*, *FRE7*, *FIT2*, *HMX1*, *FRE6*, *FRE3*, *FRE4*, *FRE2*, *ARN1*, *ARN2*) and copper ion import (*FRE7*, *FRE6*, *FRE3*, *FRE4*, *FRE2*) displayed significantly elevated levels of expression when compared to the transcriptomic response of the cells grown in the absence of doxorubicin. Furthermore, several genes involved in meiotic DNA double-strand break processing (*EXO1*, *SGS1*, *SPO11*, *RAD50*), double strand break formation (*REC102*, *MEI4*, *REC104*, *SPO11*, *RAD50*) and autophagy, CVT and mitophagy (*ATG22*, *ATG23*, *ATG 29*, *ATG36*, *ATG39*, *ATG11*, *MON1*) were up-regulated. The higher expression levels of *SRX1*, *FRE7*, *SGS1*, *REC104* and *ATG39* were further confirmed by RT-qPCR analysis (Supplementary Figs S2 and S3). Several structural and functional mitochondrial genes were also induced in yeast cells in the presence of doxorubicin.

A total of 303 genes were identified displaying significantly and differentially down-regulated expression levels when compared to control. The significantly associated GO biological process terms (Benjamini-Hochberg corrected p value < 0.05) with 292 down-regulated genes, excluding 11 genes encoding hypothetical proteins, were determined (Supplementary Table S4). The genes which were involved in amino acid biosynthesis, including the synthesis of lysine, methionine, arginine and isoleucine were observed to be down-regulated in response to the exposure of yeast cells to doxorubicin. Biological processes such as translation, glycolysis, sulfate assimilation, purine nucleotide biosynthesis, one-carbon metabolism and *de novo* IMP biosynthesis were also found to be affected in similar manner (Fig. 2). A total of 34 genes collected under a common translation term consist of the genes involved in ribosome biogenesis, ribosomal small subunit biogenesis and assembly and RNA processing. Analysis of the affected pathways indicated that biosynthesis of amino acids, glycolysis and gluconeogenesis, ribosome, sulfur metabolism were significantly repressed. A total of 10 transcription factors (TF) were identified as significantly down-regulated. Half of these TFs were involved in chromatin re-organization, restriction, silencing, nucleosome re-modeling and positioning (*ABF1*, *TBF1*, *RAP1*, *CBF1*, *NHP6A*).

Furthermore, the manual inspection of this group of genes indicated that the genes (*HHT1*, *HTA2*, *HAT2*, *HTA1*, *HTB2*, *HTB1*) involved in chromatin assembly and disassembly displayed significantly lower expression levels in response to doxorubicin exposure when compared to control. Several genes involved in the biosynthesis of cysteine, valine, leucine and arginine were also found to be significantly and differentially down-regulated. The genes involved in glutamine biosynthesis (*HIS7*, *ADE6*, *ASN1*, *URA2*, *ADE4*), NAD and *de novo* NAD biosynthesis from tryptophan (*BNA7*, *BNA5*, *NMA1*, *BNA4*), NADH oxidation (*GPD1*, *ADH1*, *ADH5*) and global nucleotide excision repair (*HHT1*, *DOT1*, *ABF1*) were repressed. The lower expression levels of *HTB2*, *BNA4*, and *ADH5* were further confirmed by RT-qPCR analysis (Supplementary Figs S2, S3).

The independent measurements of gene expression and intracellular fluxes were consistent as implied by the common GO terms (Fig. 2, indicated by *). These two different types of -omics measurements revealed some common processes that had decreased flux and transcript levels in response to doxorubicin.

### Modular analysis of differentially active sub-network

**Figure 3:**
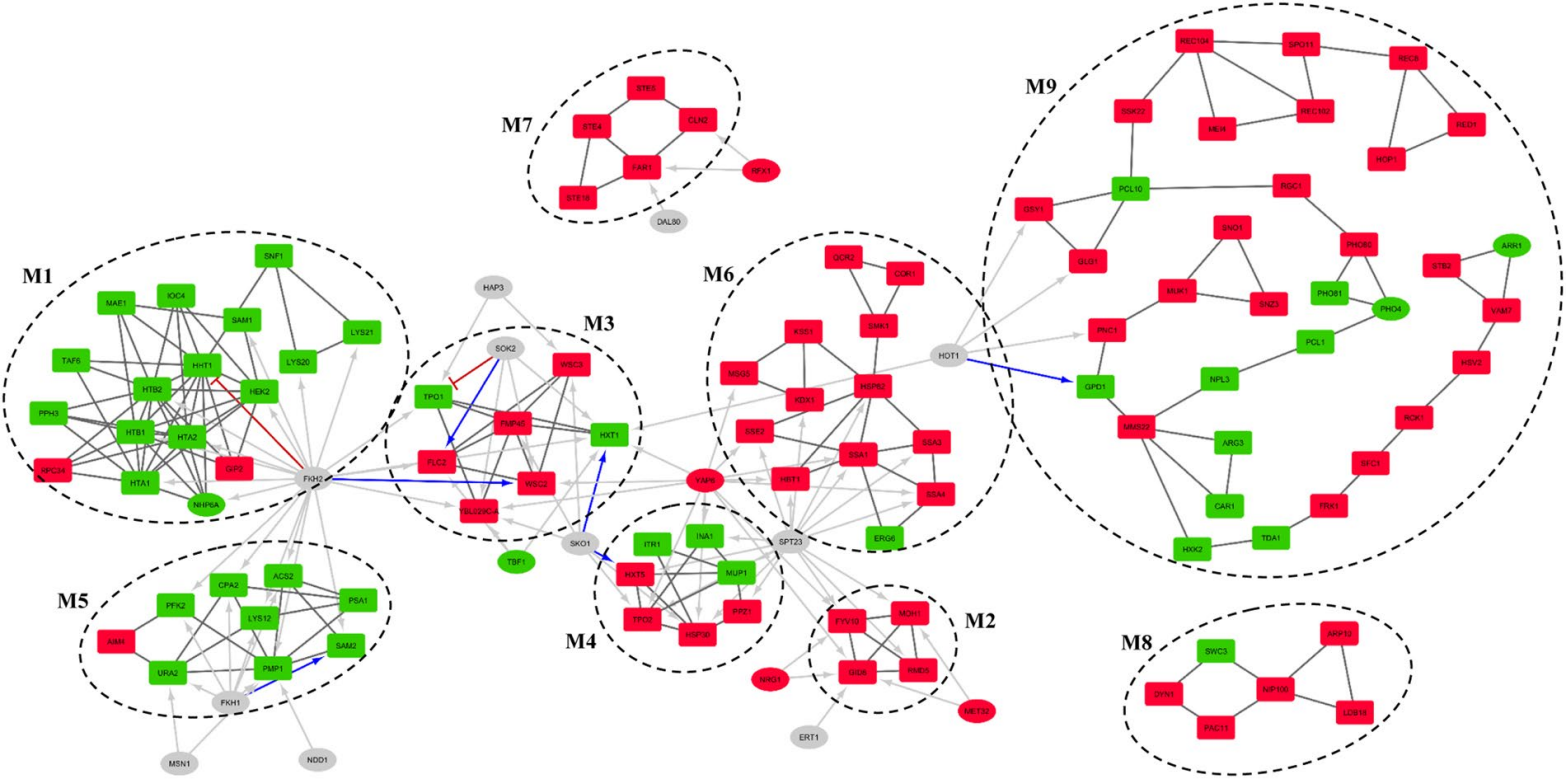
Modular organization of differentially active regulatory network (DEGRN). Nine modules identified by MCODE by using physical interaction network of DEGs (DEGN). Red nodes are up-regulated; green nodes are down-regulated while grey nodes are TFs that are not DEGs. Black connections represent physical interactions, blue arrows show positive regulation, red tapped arrows indicate inhibition, and grey arrows represent unspecified regulation. Rectangular nodes are genes/proteins while elliptic nodes are TFs. Regulatory interactions shown only for significant TFs while some TFs reside in modules were shown with only physical interactions. Inter-modular physical interactions are not shown.

Differential expression analysis of transcriptomic data collected after long-term doxorubicin treatment of yeast cells in a batch environment revealed 785 significantly induced and 292 inhibited DEGs. A differential active gene network (DEGN) was constructed using differentially expressed genes and protein-protein interaction data. Modular topological analysis of DEGN, consisting of 896 proteins and 2291 interactions, by MCODE indicated the presence of 9 densely connected modules, which are significantly associated with distinct biological processes or having nodes with similar biological roles (Supplementary Table S6 and Fig. 3). Transcription factors of which target genes were over-represented in each module were identified to determine the plausible co-regulation using TF enrichment analysis.

Fkh2p, which is involved in chromatin remodeling and a negative role on chromatin silencing, was found to be significantly associated with M1, M5 and M3. The genes in M1 is mostly down-regulated (15 out of 17) in the presence of doxorubicin and significantly associated with chromatin assembly/disassembly. Four members of the modules also have roles in DNA repair mechanism. The second module with significantly enriched targets of Fkh2p, M5, consists of mostly down-regulated (8 out of 9) genes and was found not to be associated with any GO biological process term but was significantly associated with general metabolic pathways term. Manual investigation of the constituents indicated that most of the genes of this module is involved in amino acid biosynthesis. This module was also found to be significantly associated with Fhk1p, involved in chromatin remodeling, mitotic transcription regulation, transcription termination, mating-type switching, and pseudohyphal growth, Ndd1p, involved in the positive regulation of G2/M mitotic cell cycle and Msn1p, involved in the positive regulation of transcription in response to glucose limitation.

The third co-regulated module by Fhk2p, M3, has five out of seven genes up-regulated. Although no significant GO term or pathway association could be detected for this module, manual investigation indicated that the genes in this module were involved in stress response, signal transduction, trans-membrane transport activity and cell wall organization. *YBL029C-A*, which is a protein with unknown function, is known to be localized to cell peripherals and induced with DNA replication stress. Hot1p which has stress responsive regulatory role, the down-regulated transcription factor Hap3p which is a global regulator of respiratory gene expression, Tbf1p which is involved in the negative regulation of chromatin silencing and required for full snoRNA expression, Sok2p which plays a regulatory role in PKA signal transduction were found to have significantly enriched target genes in this module. M3 seems to be co-regulated with M4 by Sko1p, which is involved in stress response. TF enrichment analysis indicates that M3, M4 and M6 were co-regulated by the stress responsive factor Yap6p. The gene encoding Yap6p is also found to be significantly up-regulated in the presence of doxorubicin in the present study, also confirmed by RT-qPCR analysis (Supplementary Figs S2 and S3). TF enrichment analysis indicated that M4 is also co-regulated with M6 and M2 by Spt23p.

The smallest module M2 consists of 4 up-regulated DEGs and significant association with proteasome mediated ubiquitin dependent protein catabolism and gluconeogenesis biological process terms accordingly. Three proteins of this module are subunits of Gid complex and the forth node, *MOH1*, is a protein with unknown function and has interactions with all other three nodes. Three regulators of this module, Yap6p, Met32p and Nrg1p, are also up-regulated DEGs themselves. Yap6p and Nrg1p are stress response factors known to be Tup1-Ssn6 complex recruiters in response to changing environmental conditions^30^. Nrg1p is involved in glucose repression and glucose sensing and is a DNA binding cofactor that target Tup1-Ssn6 complex to starch degrading genes. Met32p is involved in the biosynthesis of methionine and Ert1p is involved in the regulation of gluconeogenesis and fermentable carbon utilization. The only significant TF with direct role in protein catabolism is Spt23p, which was shown to be activated by a mechanism that involves ubiquitin/proteasome-dependent processing^31^.

The module M6, with 12 genes up-regulated out of 13, was found to be significantly associated with protein folding/refolding, SRP-dependent co-translational protein targeting to membrane GO biological process terms and protein processing in ER pathway.

The largest module M9 with 34 nodes (23 out of 34 up-regulated) has a long and branched pathway-like topology (Fig. 3). This module was significantly associated with GO biological process terms, namely meiotic cell cycle, meiotic double strand break formation and reciprocal meiotic recombination. Manual investigation of the nodes revealed that the top part, which is formed by seven up-regulated nodes, is related to meiotic cell cycle. This part of the module is connected to the rest of the module via *SSK22*, which encodes a signaling protein. The following part (*GSY1*, *GLY1*, *PCL10*, and *RGC1*) has various roles in glycogen biosynthetic process. The next part has stress responsive nodes (*PHO80*, *PHO81*, *PHO4*, *PCL1*, *NPL3*, and *MMS22*). Especially *MMS22* has an important role in double strand break repair and named after its MMS sensitivity. The module diverges into three branches on *MMS22*; the smallest branch includes down-regulated urea cycle related *ARG3* and *CAR1*, the second branch has nodes with various biological roles and ends with two up-regulated nodes, which have roles in vitamin B6 biosynthetic process (*SNO1* and *SNZ3*). The last and the longest branch has up-regulated autophagy related nodes, *HSV2* and *VAM7*. This last part of the module also has nodes with no clear biological roles (*TDA1*, *FRK1* and *STB2*). The only significant TF identified to be regulating this module is Hot1p, which targets nodes involved in glycogen and glycerol synthesis.

All five nodes of disconnected module M7 are up-regulated and all of them related to pheromone dependent MAPK signal transduction pathway leading to cell cycle arrest. Significantly up-regulated Rfx1p and Dal80p, which is a negative regulator of genes in multiple nitrogen degradation pathways, are significant TFs identified as co-regulators of M7.

There is no significant co-regulator for M8 which is significantly associated with phagosome pathway and establishment of mitotic spindle orientation and localization processes. Except from *SWC3*, which is a protein of unknown function, all nodes of the module are up-regulated. All up-regulated gene products have indirect roles in mitosis via dynein activity. Induction of these genes together with induced cell cycle arrest may imply that doxorubicin leads to organelle re-organization or nuclear instability as they are the additional roles of these nodes.

## Discussion

Although the molecular mechanism of doxorubicin is not clear, two mechanisms (i) intercalation into DNA and inhibition of topoisomerase II leading to changes in chromatin structure and (ii) generation of free radicals and oxidative damage to biomolecules have been proposed as potential mode of action^32^. In order to shed a light on the underlying molecular mechanism of doxorubicin, we have used a systems based integrative approach in the present study. The genome-wide transcriptional response of yeast cells to long-term exposure to doxorubicin and the changes in fluxes identified using a genome scale model were comparatively analysed. Transcriptional re-wiring of the metabolism was also investigated by modular analysis of active sub-network via integrating transcriptome and interactome, and TF enrichment analysis.

Genes involved in response to oxidative stress were found to be significantly induced in the transcriptomic response of yeast cells to long-term exposure to doxorubicin, as expected. Although low levels of ROS is responsible for the survival and proliferation, induction of ROS levels is associated with tumorigenesis. However, a further increase may lead to apoptotic, autophagic, ferrotopic and necrotic cell death. Cancer cells have higher ROS levels than normal cells, which make them more susceptible to ROS inducing chemotherapeutic agents or radiation therapy than non-cancerous cells^12,33^.

**Figure 4:**
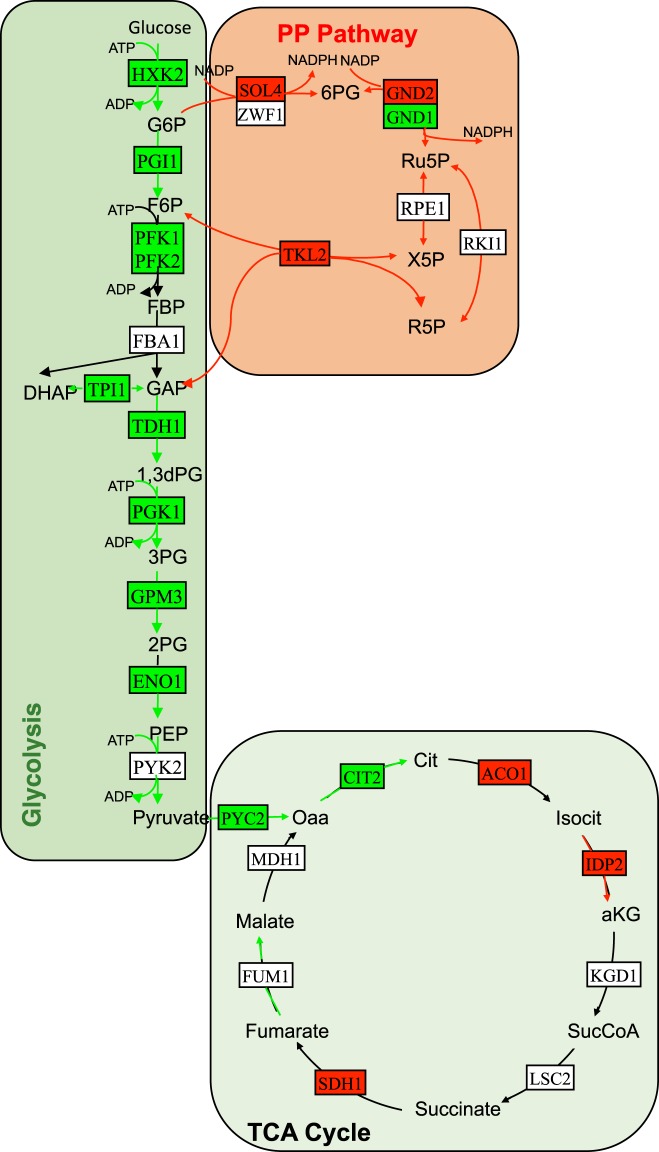
Integrative analysis of central carbon metabolism in the presence of doxorubicin. Red and green arrows indicate increased and decreased fluxes, respectively. Red and green rectangles indicate up- and down-regulated genes, respectively.

PP pathway implicated in the synthesis of biological macromolecules and NADPH was found to be up-regulated (Fig. 4). NADPH creates a reducing environment, and reduces glutathione to prevent the oxidative stress^34^. Several genes involved in *de novo* NAD biosynthesis from tryptophan and NADH oxidation, were down-regulated. This observation is consistent with the work of Davies and Doroshow^35^, in which inhibition of the reduction of NAD^+^ to NADH by doxorubicin was reported. In another study, it was shown that NADH oxidase activity was inhibited by doxorubicin when exposed to mice^36^.

Transcriptional level of the genes and fluxes throughout the glycolytic pathway were observed to be down-regulated (Fig. 4). An up-regulation of glycolysis and glucose uptake are the prominent features of cancer cells. Therefore, this pathway has been considered as a target for the development of new therapeutics^37^.

Sulfate assimilation pathway and expression levels of several genes involved in glutamine biosynthesis were repressed in response to the long-term presence of doxorubicin. Sulfate assimilation pathway is also closely linked to the glutathione production^38^. A decrease in the activity of this pathway might possibly result in a reduction at the level of glutathione, which is an electron donor in ROS detoxification and oxidative stress protection in ER.

**Figure 5:**
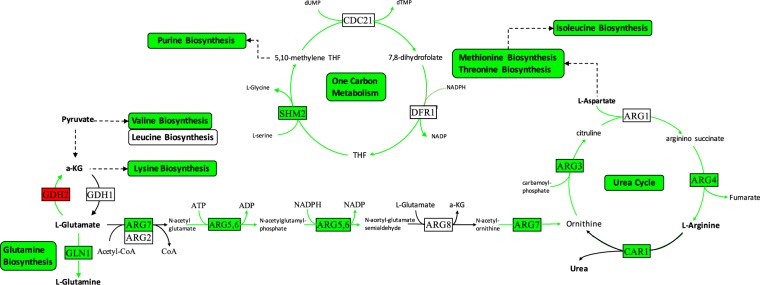
Integrative analysis of down-regulated pathways in response to doxorubicin. Green arrows indicate decreased fluxes. Red and green rectangles indicate up- and down-regulated genes, respectively.

The system-wide integrated approach used in this study indicated a pronounced re-wiring of the metabolism to cope with the stress caused by this chemical. In addition to glycolysis and sulphur assimilation, several pathways related to ribosome biogenesis/translation, nucleotide biosynthesis, and *de novo* IMP biosynthesis and one-carbon metabolism were observed to be repressed in response to doxorubicin (Fig. 5). One-carbon metabolism is involved in the synthesis of essential biomolecules such as nucleotides and amino acids as well as in epigenetic modifications. The up-regulation of this latter process in cancer has been reported due possibly to the increasing need of building blocks in proliferative cells^39^. Therapeutic agents that block one-carbon metabolism are found to be effective in cancer therapy^39–41^. However, the toxicity of these therapeutic agents to non-cancer cells created an obstacle for their widespread use.

The biosynthesis of several amino acids including the synthesis of lysine, methionine, arginine and isoleucine were observed to be significantly down-regulated in response to the exposure of yeast cells to doxorubicin. These results are in good correlation with the findings that the supplementation of single amino acid in minimal media protected yeast cells from doxorubicin toxicity^42^. The amino acids that showed highest protection were associated with TCA cycle.

Sterol/steroid biosynthesis pathways were found to be repressed in the presence of doxorubicin according to the analysis on differential fluxes, but not based on transcriptomic measurements. It has been shown that cancerous cells depict specific changes in various parts of lipid metabolism^43^, with some causing lipid metabolic reprogramming in cancer metastasis^44,45^, sterol molecules having roles in the regulation of cellular proliferation in cancer^46^.

Doxorubicin, as other anthracyclines, is considered to damage DNA through intercalation or direct alkylation and may result in the formation of DNA double-strand breaks^47^. DNA damage induces Rad53 checkpoint sensing and signaling pathway in yeast^48^. Several components of this pathway (*MEC1*, *RAD24*, *HUG1*) were observed to be significantly up-regulated upon long-term exposure of yeast cells to doxorubicin. The higher expression level of *HUG1* was also confirmed by RT-qPCR analysis (Supplementary Figs S2 and S3). Detailed investigation of the transcriptional response indicated that the genes involved in cellular response to DNA damage and DNA repair were also induced. Several genes functioning in DNA repair were observed to be up-regulated upon doxorubicin exposure^49^. Modular analysis of DEGN also indicated that a module is significantly associated with nucleosome assembly/disassembly and several genes within this module was also found to be involved in DNA repair. Major transcriptional repressor of DNA damage regulated genes, *RFX1*, which recruits Tup1p/Cyc8p to the promoters of these genes, was found to be induced. The mammalian homolog of this gene, *RFX-1*, was shown to regulate several genes involved in immunity and cancer progression^50,51^. It has been reported that intercalation of doxorubicin causes torsional stress leading to the nucleosome destabilization results in anthracycline-mediated cell killing^52^.

Several copper import genes were also found to be induced within the framework of this study. Furthermore, *CUP2*, which is involved in the activation of the metallothionein genes in response to elevated copper levels and required for the regulation of copper genes in response to DNA damaging reagents, was observed to be significantly up-regulated in response to doxorubicin. A regulatory relationship between DNA damage and copper response in yeast was proposed through a genome-wide genetic screening and the up-regulation of copper import genes in response to DNA-damaging agents^53^. Although a detailed study is required to fully understand this relationship, it was proposed that Mac1p may exist in different redox forms in the cell^53^.

Meiosis and sporulation were observed to be significantly induced in response of yeast cells exposed to doxorubicin. Meiosis was shown to be up-regulated in the presence of doxorubicin in yeast cells^49^. A deficiency in glucose and nitrogen are known to induce meiosis followed G1 arrest of the mitotic cell cycle in diploid yeast cells^54,55^. When yeast cells starve for essential nutrients, sporulation and meiosis coupled with spore formation is induced^56^. However, absence of any nutritional deficiency in our experimental system indicates that G1 arrest and induction of meiosis are triggered by doxorubicin, which may cause a misperception in glucose sensing. Several transcription factors implicated in the regulation of cellular response to nutrient levels and general stress were also identified among the significantly up-regulated genes. The up-regulation of these biological processes in yeast cells subjected to long-term exposure to doxorubicin, indicates that the yeast cells experience a misperception of the nutritional environment in the presence of doxorubicin. Furthermore, several members of Snf3/Rgt2/Rgt1 (SSR) glucose sensing and signaling pathway and consequently hexose transporter genes were found to be significantly up-regulated to cope with this misperception.

Induction of carbohydrate metabolic process in the presence of doxorubicin reflects the up-regulation of several genes involved in the catabolism of galactose and maltose, as well as PP pathway and degradation of yeast storage carbohydrates glycogen and trehalose, which are known to be produced under glucose starvation conditions. This finding agrees well with a possible misperception in glucose availability.

The expression level of *SNF1*, the mammalian ortholog of the AMP-activated protein kinase (AMPK) involved in the regulation of cellular energy homeostasis and metabolism as well as in glucose sensing, was found to be significantly repressed in the present study. Inhibition of SNF1 in response to a DNA damaging agent, MMS, accompanied with a switch from respiration to fermentation is also reported in yeast cells^57^. Inhibition of AMPK in several non-carcinoma cells in response to doxorubicin have also been observed and a pre-activation of this pathway has been suggested to prevent doxorubicin-induced cell death and cardiotoxicity^58–61^. Both pro- and anti-tumorigenic roles of inhibitors and activators of AMPK in cancer therapy have been suggested^62^.

MAPK signaling pathway was found to be significantly induced in response to stress caused by the presence of doxorubicin. MAPK pathways are functional tools to adapt cells to stress by targeting a wide variety of downstream effectors including transcriptional factors^63^. The induction of MAPK pathway by anthracycline based anti-tumor antibiotics has been reported in neuroblastoma cells^64^, rat hepatoma cells^65^, human cervical carcinoma cells^66^, monoblasts^67^, glioblastoma cells^68^ and, in a time and dose dependent manner, in breast epithelial and carcinoma cells^69,70^. A dual role of MAPK/ERK pathway as a tumor suppressor and a pro-oncogenic signal has been well recognized^71^. Therefore, it should be noted that the understanding of the effect of doxorubicin on MAPK in each tumor type specific microenvironment is very important in designing single or combination therapies with doxorubicin.

Modular analysis of differentially active sub-networks revealed nine modules and investigation of transcriptional regulators of these modules indicates that Yap6p, Spt23p and Fkh2p are the most active transcriptional factors that each co-regulates more than two modules. Yap6p physically interacts with the Tup1-Cyc8 complex and recruits Tup1p, which is a general repressor of transcription, to its targets. Expression of this stress responsive TF is also up-regulated and its role in DNA damage was reported to be through chromatin remodeling^72^. Fkh2p has a major role in cell-cycle regulation and also has a role in chromatin remodeling. Its targets in the differentially active sub-network are mostly down-regulated in contrast to the targets of Yap6p and Spt23p. Targets of Spt23 in the network are related to degradation of enzymes required for gluconeogenesis. Down-regulation of gluconeogenesis is suggested as an attractive therapeutic strategy in recent cancer research^73^ and repression of gluconeogenesis through Spt23p can be considered as one of the anti-cancer effects of doxorubicin.

A genome-wide screen of *S*. *cerevisiae* diploid deletion collection resulted in identification of 376 resistance genes to doxorubicin^29^. Among those 376 genes, 43 (22 up, 21 down) were found to be differentially expressed in the presence of doxorubicin in the present study. 22 up-regulated genes were found to be enriched (p value < 0.05) in cell cycle, cellular response to DNA damage stimulus, DNA repair and DNA topological change terms. Among those, *TOP3*, *RAD50* and *RAD54* have roles in the homologous recombination pathway. Within the 21 down-regulated genes, genes involved in glycolysis, pentose phosphate pathway and purine biosynthesis were observed.

Unique mode of action of several ROS causing or DNA damaging agents were already reported. A comparative analysis indicated that exposure to doxorubicin triggers unique transcriptional response. Only less than 15% of DEGs that are sensitive to doxorubicin are common with DEGs identified as sensitive to DNA damaging agents; MMS and ionizing radiation^74^. The results of a study using cumene hydroperoxide was compared with a previous study where hydrogen peroxide was used as source of oxidative stress^75,76^. When those are compared with the results of the present study, it was observed that among the genes up-regulated in CHP and H_2_O_2_-induced stress, 56 out of 162 were found to be up-regulated in the present study. And among the genes down-regulated in CHP and H_2_O_2_-induced stress, 53 out of 481 were found to be down-regulated in the presence of doxorubicin. These variations in transcriptional response reveal that despite common expression patterns, larger part of the transcriptome reacts differently and production of ROS causing oxidative stress does not have a universal mechanism. Focusing on disparate results will guide us on understanding specific mechanisms of the drugs on cellular phenomena.

The intercalation of doxorubicin into DNA and inhibition of topoisomerase II leading to changes in chromatin structure has been proposed as one of the major effects of doxorubicin. We have compared the differentially expressed genes identified in the present study with genes specifically up- and down-regulated upon 10 min inactivation of *TOP2*, excluding the environmental stress response genes, in yeast cells^77^. 11 genes out of 97, identified to be selectively inhibited by the inactivation *TOP2*, were also observed to be down-regulated in the present study. Among the 173 selectively up-regulated genes by the inactivation of *TOP2*, 61 genes were also found to be up-regulated in the presence of doxorubicin in the present study. Despite requiring further investigation, the incidence of 61 common genes might be an indicator for the inactivation of *TOP2*, which is one of the important targets for anti-cancer drugs^78^, under doxorubicin exposure.

In addition to the generation of ROS and DNA damage, the present study indicates that doxorubicin induces an impressive re-wiring of metabolic and signaling pathways, which are already known or suggested as therapeutic targets in cancer. The changes, which are induced, are in the direction to alleviate the changes occurring in cancerous cells.

Further studies including time dependent response of yeast cells to different concentrations of doxorubicin and integration of transcriptomic response with metabolomics, proteomics and phosphoproteomics will be required to dissect the specific response of yeast cells to doxorubicin and its targets in the treatment of cancer. It should be noted that adaptation of yeast cells to the long-term presence of doxorubicin needs also to be considered. Future research on the genomic re-organizations and dynamic re-organization of the response at different -omics levels in the presence of different concentrations of doxorubicin would give further insight into the molecular mechanisms underlying the doxorubicin treatment and complement the present study. Although the essential central pathways were conserved among yeast and human, detailed studies in normal cells and tumor microenvironment will be required to elucidate the complex cellular mechanisms of this and other therapeutic agents.

## Materials and Methods

### Strain and preculture conditions

Homozygous *ho*∆/*ho*∆ strain of *S*. *cerevisiae* diploid BY4743 (*MATa/MATΔ his3Δ1/his3Δ1 leu2Δ0/leu2Δ0 lys2Δ0/*+ *met15Δ0*/+ *ura3Δ0/ura3Δ0*) was kindly provided by Prof. Stephen G. Oliver. Cells were grown to stationary phase in shake flasks in YPD medium. Culture aliquots containing 50% (v/v) glycerol were kept at −80 °C until further use.

Precultures were grown in F1 minimal medium^79^ at 30 °C and 180 rpm overnight. F1 minimal medium had the following composition per liter: 20 g glucose, 3.13 g (NH_4_)_2_SO_4_, 2.0 g KH_2_PO_4_, 0.55 g MgSO_4_.7H_2_O, 0.1 g NaCl, 0.02 g uracil, 0.02 g L-histidine, 0.1 g L-lucine, 90 mg CaCl_2_.H_2_O, 70 µg ZnSO_4_.7H_2_O, 50 µg FeCl_3_.6H_2_O, 10 µg, CuSO_4_.5H_2_O, 10 µg H_3_BO_3_, 10 µg KI, 62 mg inositol, 14 mg thiamine.HCl, 4 mg pyridoxine, 4 mg Ca-pantothenate, and 0.3 mg biotin.

### Shake flask experiments

To decide on the most appropriate drug concentration, *S*. *cerevisiae* cells were grown in F1 minimal media having different concentrations of doxorubicin (Sigma, Cat. No: D1515). Doxorubicin was added into the media before the inoculation of cells to have final concentration of 5, 10, 20, 50 µM. OD_600_ was followed.

### Bioreactor experiments

Aerobic batch cultures were grown in F1 minimal medium (with 20 g/L glucose) in 2 L B-Braun Biostat B Plus bioreactors with a working volume of 1.5 L (Sartorius Stedim Systems GmbH, Melsungen, Germany). The cultivation temperature was controlled at 30 °C, and the pH was controlled at 5.5 with 1 M NaOH and 1 M HCl. The bioreactor was operated at a stirrer speed of 800 rpm and an aeration rate of 1.5 L/min. Under these conditions, oxygen transfer was sufficient because the dissolved oxygen never dropped below 85% of air saturation that was measured online with a DO sensor (VISIFERM DO 225, Hamilton Bonaduz AG, Switzerland). A silicone antifoam agent (Sigma-Aldrich, USA) was diluted 1:10 (v:v) and intermittently added to the medium. During the experiments, the dissolved oxygen tension, pH, temperature, and the carbon dioxide and oxygen in offgas were monitored online with a gas analyzer (BlueSens, Herten, Germany).

Control cultures were grown solely in F1 media, cultures with doxorubicin additionally contained 20 µM doxorubicin, added before the inoculation of cells. Cells were subjected to doxorubicin exposure approximately for 50 hours before the sample collection. Each fermentation experiment in bioreactor (control and with doxorubicin) was performed in triplicate.

Samples were collected at the mid-exponential phase at an OD_600_ of 0.6–0.7 for analysis of biomass, RNA and metabolites. The samples for RNA and metabolite analysis were immediately frozen in liquid nitrogen and stored at −80 °C until further processing.

### Measurement of cell dry weight and analysis of extracellular metabolites

The cell dry weight was obtained gravimetrically: 4 × 1 mL broth was centrifuged (8000 rpm, 6 min) in previously weighed Eppendorf tubes and the cells were washed twice with demineralized water. The Eppendorf tubes containing cell pellets were dried in an oven at 70 °C for 48 h until constant weight.

The supernatants obtained by the centrifugation of the broth samples were used for analysis of extracellular metabolites. The concentrations of glucose, glycerol and ethanol in these samples were analyzed enzymatically according to the manufacturer’s instructions (Boehringer Mannheim/R-Biopharm, Darmstadt, Germany).

### RNA extraction and microarray experiments

The control group and doxorubicin treated yeast cells were derived from three independent batch cultures grown in bioreactors as explained above. RNA extraction was carried out with QIAcube (Qiagen, USA) using the enzymatic lysis protocol (Qiagen RNeasy mini kit; Cat no: 74106). The quality and quantity were checked via UV–vis spectrophotometer (NanoDrop ND-1000, Thermo Fisher Scientific Inc., USA). RNA integrity number values were obtained using a microfluidics-based platform (Bioanalyzer 2100 Agilent Technologies, USA) and samples with RIN values 7–10 were processed for microarray analysis. All protocols were used according to their individual directions supplied by the manufacturer.

Microarray experiments were carried out on Yeast 2.0 Arrays, using GeneChip® 3′ IVT Express Kit for cDNA preparation and amplification (Affymetrix Inc., USA). Hybridization, wash and stain was carried out as described previously^80^. All the kit protocols were used as described by the manufacturer.

### Microarray data acquisition and analysis

The data was processed using MATLAB Bioinformatics Toolbox. The datasets were normalized by Robust Multi-Array Average (RMA) via ‘affyrma’ command. The significance of gene expression was evaluated using unpaired t-test with a p-value threshold of 0.05 and fold change >1.5, and differentially expressed genes (DEGs) were identified. GO term enrichments were carried out in DAVID^81^ (Benjamini-Hochberg corrected p value < 0.05) after excluding the genes with unknown function from each up- and down-regulated gene sets.

### RT-qPCR experiments

The expression level of *RFX1*, *CUP2*, *PDR8*, *SRX1*, *FRE7*, *SGS1*, *REC104*, *ATG39*, *YAP6*, *HUG1*, *NHP6A*, *HTB2*, *BNA4*, *ADH5*, *ENO1*, *SHM2* and *ARG4* were determined in the cells grown as control and in the presence of doxorubicin. *FBA1* was used as the housekeeping gene^82^.

The primers were designed using Roche Array Design Centre with the default settings (see Supplementary Table S7). The amplicon sizes were around 60 bp. Initial concentration of RNA in all samples were set to be 50 ng/μl. Reverse transcription was performed using BioRad iScript cDNA Synthesis Kit, as described by the manufacturer, in instrument Applied Biosystems 2720 Thermal Cycler (USA). Real-time RT-qPCR with the cDNAs was then carried out with SensiFAST SYBR No-ROX Kit as described by the manufacturer (Bioline Reagents Ltd, UK, Cat no: BIO-98020). The PCR reactions were performed in a final reaction volume of 20 μl containing the final concentration of 0.5 µM of forward and reverse primers in Roche LightCycler 96 instrument (Roche Diagnostics GmbH, Mannheim, Germany). 96-well microplates and adhesive sealing films were manufactured by Axygen (USA).

Quantification cycle (Cq) values were called using the LightCycler 96 Application software yielding amplification plots. Relative gene expression values were calculated from Cq values using ΔΔCq method.

### Metabolic flux analysis

Flux balance analysis (FBA), a common approach used to calculate the flow of metabolites through a network of biochemical reactions, was carried out using Yeast7.0 genome-scale metabolic model, developed for *S*. *cerevisiae*^83^. This model contains all the known biochemical reactions and the genes related to those. FBA is based on mass balance equations for each intracellular metabolite $({\bf{x}})\,{\rm{as}}\,\frac{{\rm{d}}{\bf{x}}}{{\rm{dt}}}={\bf{S}}.{\bf{v}}$. In the stoichiometric matrix **S** (*mxn*) with m number of metabolites and n number of reactions, entries are the stoichiometric coefficients for the corresponding biochemical reaction and **v** (*nx1*) is the rate vector of the model. At (pseudo) steady-state $(\frac{{\rm{d}}{\bf{x}}}{{\rm{dt}}}={\bf{0}})$ an algebraic set of equations (**S**.**v** = 0) is obtained. FBA allows the calculation of intracellular fluxes of each reaction as a function of experimentally measured extracellular fluxes. In a typical case where *n* > *m*, the system is underdetermined, yet a unique solution can still be obtained under biologically relevant optimality criteria. Overall, when solved with a given a set of upper and lower bounds on **v**, FBA results in a distribution of metabolic fluxes, which minimize or maximize an objective function^84^. The biomass specific rates of glucose consumption, glycerol and ethanol productions were calculated from the respective mass balances for the liquid phase. These uptake/production rates were used as constraints. Alternate optima were eliminated to obtain a unique set of flux distributions, by applying flux variability analysis^85^. The resulting genome scale model being under-determined was optimized for maximum ATP hydrolysis. All calculations were performed using MATLAB and COBRA toolbox^86^, for each experiment individually (in total six cases).

Different fluxes between cultures grown in the absence and presence of doxorubicin were identified based on the fold change (down: <0.8, up: >1.10).

### Integration of fluxome and transcriptome

To find out the functional annotation biological process GO terms of the reactions, which have different flux values between two conditions, the genes associated with these reactions were identified. These gene sets were then used in GO-term enrichment analysis, carried out in DAVID^81^ (Benjamini-Hochberg corrected p value < 0.05).

### Modular analysis of active network

An active network, consisting of 915 proteins with 2301 connections, was constructed using DEGs and protein-protein interactions extracted from BIOGRID v.3.4.146. After removing the disconnected residues, the connected active protein-protein interaction network (DEGN) with 896 nodes and 2291 edges was identified.

DEGN was visualized by using Cytoscape v.3.5.1 and densely connected modules were extracted by using MCODE application of Cytoscape^87,88^. Default settings of MCODE were applied (haircut: on; fluff: off). Minimum number of nodes was set to be four.

In order to characterize biological significance of nine modules identified by MCODE, significantly associated (Benjamini-Hochberg corrected p value < 0.05) gene ontology terms and pathways to each module were identified by using DAVID 6.8 functional annotation tool^89^.

Transcription factors significantly associated to the modules (Bonferroni corrected p value < 0.01) were identified using YEASTRACT 2017^90^. ‘Only DNA-binding evidence’ was used for regulatory interactions. Resulting regulatory network of densely connected modules of DEGs (DEGRN) was visualized by using Cytoscape v.3.5.1.

## Electronic supplementary material

Supplementary Dataset 1

Supplementary Dataset 7

Supplementary Dataset 2

Supplementary Dataset 3

Supplementary Dataset 4

Supplementary Dataset 5

Supplementary Dataset 6

## Data Availability

The microarray data has been submitted to ArrayExpress at the European Bioinformatics Institute under accession number [E-MTAB-6634] in compliance with MIAME guidelines.
