# Supplementary material for: Doxorubicin induces an extensive transcriptional and metabolic rewiring in yeast cells
Source: Sci Rep. 2018 Sep 12;8:13672. doi: 10.1038/s41598-018-31939-9 (PMC6135803; doi:10.1038/s41598-018-31939-9)
Supplement: Supplementary file 1 — Supplementary Dataset 1 [file 41598_2018_31939_MOESM1_ESM.pdf]

Supplementary Information for

Doxorubicin induces an extensive transcriptional and metabolic rewiring in yeast cells

Hilal Taymaz-Nikerel, Muhammed Erkan Karabekmez, Serpil Eraslan, Betul Kirdar

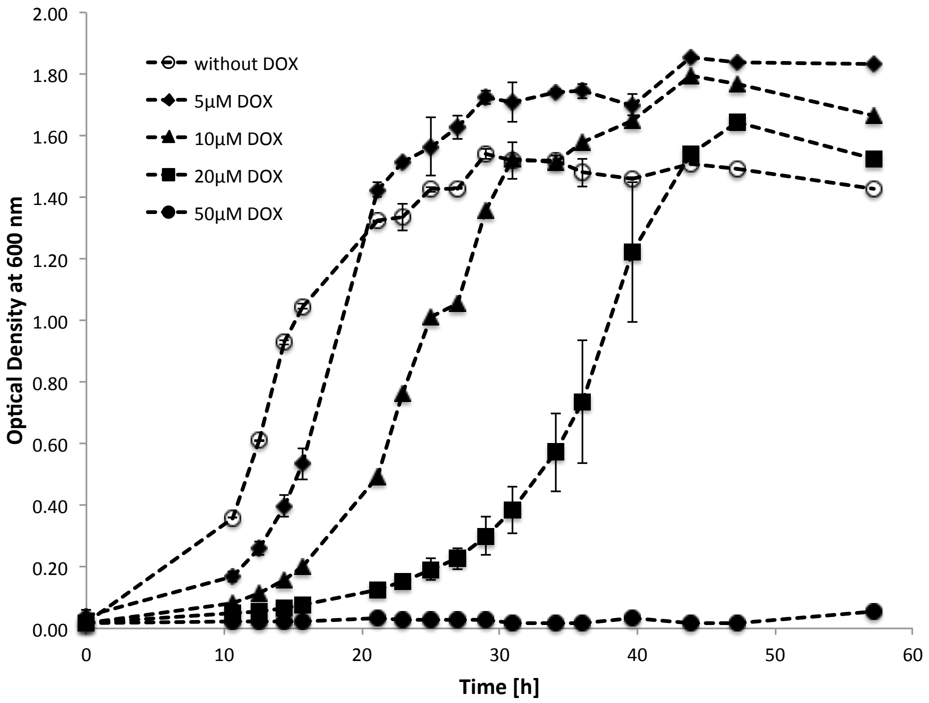

Figure S1: Growth curves with different concentrations of doxorubicin (DOX) in shake-flasks

Table S1: Uptake/secretion rates during mid-exponential phase in aerobic batch cultures of *S. cerevisiae*

	q_X [h^{-1}]	$-q_{glucose}$ [mmol $gX^{-1}h^{-1}$]	$q_{glycerol}$ [mmol $gX^{-1}h^{-1}$]	$q_{ethanol}$ [mmol $gX^{-1}h^{-1}$]
Control	0.26	11.7	1.3	18.8
	0.25	13.1	0.7	13.1
	0.28	14.4	1.3	24.6
with doxorubicin	0.11	5.1	0.7	-
	0.09	6.8	0.7	6.8
	0.11	7.5	0.5	6.2
