# Supplementary material for: Doxorubicin induces an extensive transcriptional and metabolic rewiring in yeast cells
Source: Sci Rep. 2018 Sep 12;8:13672. doi: 10.1038/s41598-018-31939-9 (PMC6135803; doi:10.1038/s41598-018-31939-9)
Supplement: Supplementary file 2 — Supplementary Dataset 7 [file 41598_2018_31939_MOESM2_ESM.pdf]

Supplementary Information for

Doxorubicin induces an extensive transcriptional and metabolic rewiring in yeast cells

Hilal Taymaz-Nikerel, Muhammed Erkan Karabekmez, Serpil Eraslan, Betul Kirdar

Table S7: Primer sequences of the genes analyzed in RT-qPCR

Gene	Primers		Gene	Primers	
RFX1	Left	GGCTCCCATAAACCATCTCC	*HUG1*	Left	GTCAAGACCGGCTACTTATTCC
	Right	AGGTTGCCGTTGTGTTGC		Right	ACGTTCGACGGCAATGAT
CUP2	Left	GAAATTAAGGCCGACAGCAT	*NHP6A*	Left	GGACAAGTCGGCAAGAAGTT
	Right	CTTTGCTCAAGCGGATCG		Right	TCGTAAGGCTGCTTTTCCTC
PDR8	Left	TAGAGATACCACGGACAAGAACC	*HTB2*	Left	CCAAAGCTCCAGCTGAAAAG
	Right	TCTTTTGCATTACCTTGCAAATC		Right	TACCATCGACGGAGGTTGA
SRX1	Left	TCAAGGGACAGACGCTATATTATG	*BNA4*	Left	GGAAAGGCCATCCTACTCG
	Right	GGCGGTCGTATGCCTGTA		Right	TACCTTGGCCGTAAAATGGA
FRE7	Left	CCATTCTACAGACCTTGTGCAG	*ADH5*	Left	TGGTACTTTTCAAGAATACGCAAC
	Right	ATTATGCCTGCACGAACAGA		Right	CAAGATTGACGTTTGGTGGA
SGS1	Left	AGGGGGTGGTAAATCTCTTTG	*ENO1*	Left	TGTCCCATCTGGTGCTTCTA
	Right	TGTGTTTTACCCGATTTCACC		Right	TCCACTTGGATTTGTCACCA
REC104	Left	AGTGACACATTATATGACGATCCTTT	*SHM2*	Left	CTTTGGTTCCAGGTGGTGTC
	Right	CCTCTATTTCATCTCCTGCTTGA		Right	TCACCCATTCCTCTAGTGGTC
ATG39	Left	TGCATGAAAGCTCCTTTGC	*ARG4*	Left	GCGTTGAAAGACGAGATGCT
	Right	GTGTTCGATAGGACATCTGCAT		Right	CCAATTGCTTCAATACAGCAGA
YAP6	Left	CTCCCATCTCGCAATACCA			
	Right	GCGGTATCAGCACTGTTTTG			

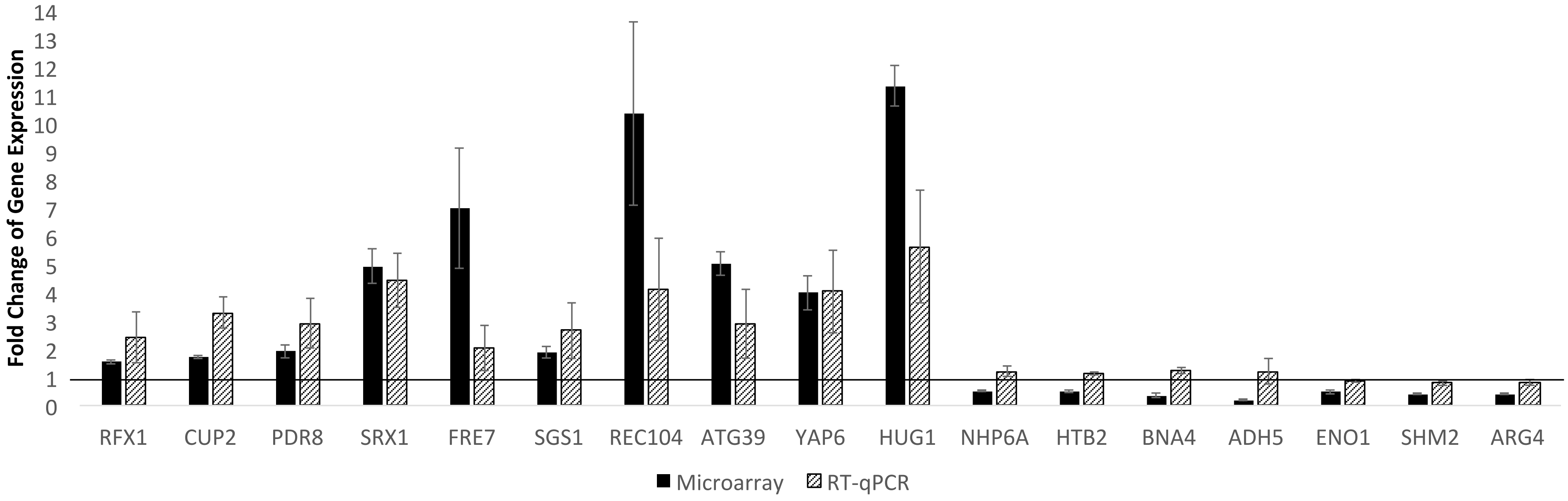

Figure S2: Comparison of fold change in the expression of selected differentially expressed genes in the presence of doxorubicin relative to control, obtained via microarray or RT-qPCR analysis. Error bars represent the standard errors.

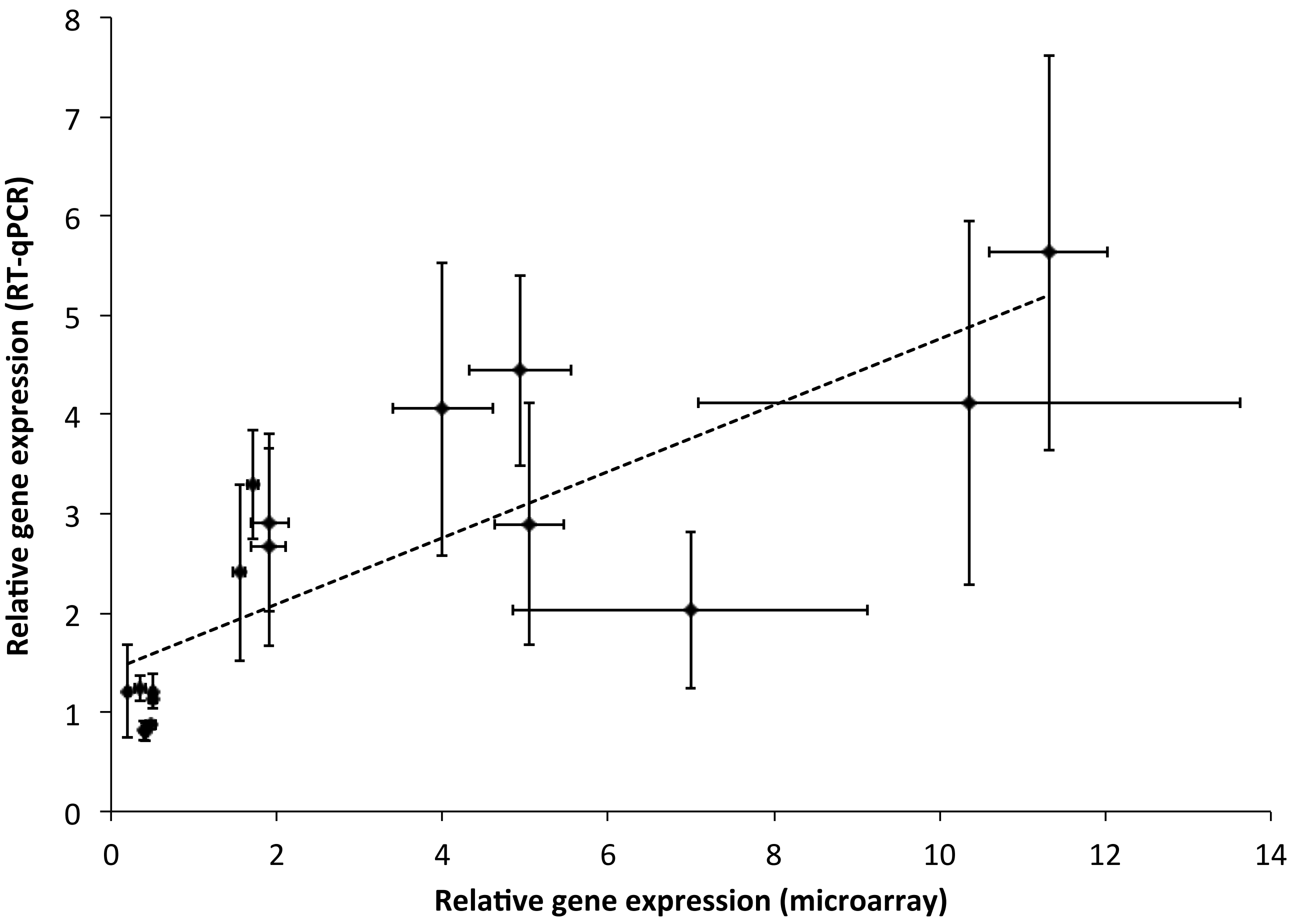

Figure S3: Correlation analysis of RT-qPCR and microarray data of selected differentially expressed genes. Line fit plot showed a significant correlation (r=0.8018, p-value=0.00011, n=17, df=15).
